# Association and Influencing Factors of Body Image Anxiety and Social Avoidance Behaviour in Children With Flat Feet

**DOI:** 10.62641/aep.2256

**Published:** 2026-10-15

**Authors:** Cheng Qian, Kuijie Zhang

**Affiliations:** ^1^Department of Orthopedics, Haiyan People's Hospital Affiliated to Jiaxing University, 314300 Jiaxing, Zhejiang, China

**Keywords:** children, flatfoot, body image, anxiety, social avoidance, correlation

## Abstract

**Background:**

This study aimed to explore the relationship between body image anxiety and social avoidance behaviour in children with flat feet, and to analyse the influencing factors associated with social avoidance behaviour.

**Methods:**

A retrospective analysis was conducted on the clinical data of 98 children with flat feet and 104 children with normal arches who visited a tertiary hospital between January 2022 and June 2025. Measurement results of the Body Image Scale (BIS), Social Anxiety Scale for Children (SASC), Children's Depression Inventory (CDI), and Self-Perception Profile for Children (SPPC) were extracted. Differences between the two groups were compared, and Spearman correlation analysis was used to analyse the relationships between variables. Binary logistic regression (with independent models constructed after collinearity diagnosis) was used to analyse the influencing factors of social avoidance behaviour.

**Results:**

The flat-foot group scored significantly higher than the normal-arch group in body image anxiety (16.36 ± 5.21 vs. 11.75 ± 4.38, *p* < 0.001) and social avoidance behaviour (10.86 ± 3.37 vs. 7.23 ± 2.96, *p* < 0.001), whereas their self-worth level was significantly lower (14.69 ± 3.33 vs. 19.21 ± 2.69, *p* < 0.001). No statistically significant difference was observed in depression levels between the two groups (*p* > 0.05). The severity of flat feet was positively correlated with body image anxiety (r = 0.886, *p* < 0.001) and social avoidance behaviour (r = 0.882, *p* < 0.001), and negatively correlated with self-worth level (r = –0.893, *p* < 0.001). Logistic regression analysis showed that the severity of flat feet (moderate: odds ratio [OR] = 2.399, severe: OR = 3.928), negative comments related to foot appearance or gait abnormalities (OR = 2.683), and body image anxiety (OR = 1.172) were risk factors for significant social avoidance behaviour in children with flat feet, whereas self-worth (OR = 0.845) was a protective factor.

**Conclusions:**

Children with flat feet exhibit significant body image anxiety and social avoidance behaviours, with body image anxiety and social avoidance behaviours being closely related. Clinical intervention should focus on the children's psychosocial adaptation alongside foot function rehabilitation, providing targeted psychological support and self-worth enhancement interventions.

## Introduction

Flatfoot is a common foot structure abnormality characterized by collapsed or missing arches, with a high incidence rate in children [1]. Epidemiological surveys show that the prevalence of flatfoot in children aged 3 to 6 years has increased to 21%–57% [2]. Most of them are physiological flat feet; however some children may have symptoms such as abnormal gait, limited motor ability or lower limb fatigue. Although the arches naturally develop and mature with age in many children, the persistent flatfoot may still have potential impacts on their physical function and psychological development [3].

In recent years, children’s mental health issues have received increasing attention. Body image, as an important part of self-concept, develops rapidly and becomes increasingly sensitive during the school-age years [4, 5]. Body image anxiety refers to the negative emotional experience of an individual due to dissatisfaction with their own appearance, body shape or physical function, which is often manifested as feelings of shame, inferiority or avoidance behaviour [6]. Foot abnormalities may not only affect athletic performance but also be a significant physiological characteristic that draws attention from peers or negative comments. Žukauskas *et al*. [7] found that even children with conventionally defined “asymptomatic” flexible flat feet experienced significant declines in health-related quality of life (especially in the domains of emotional function and social psychological function), suggesting that the impact of flat feet on children’s psychosocial adaptation may be underestimated in clinical settings. Dibello *et al*. [8] further confirmed that children with painful flexible flat feet generally experienced impaired quality of life before undergoing surgical treatment, particularly in terms of emotional function, social function, and school performance, and their families also bore the corresponding psychological burden. These studies collectively indicate that, regardless of symptom severity, as a visible physical characteristic, flatfoot may influence body image perception, thereby triggering anxiety and social avoidance tendencies, posing a potential threat to children’s psychological health development.

In addition, social avoidance behaviour, as a defence mechanism for children to cope with negative social experiences, is manifested as social withdrawal, silence or refusal to participate in group activities during social interactions [9, 10]. Previous research has demonstrated that body image anxiety is an important psychological factor associated with social avoidance in children [11]. In children with flat feet, changes in foot appearance and functional limitations may indirectly exacerbate anxiety and social avoidance tendencies by affecting body image perception. However, current research on psychosocial adaptation of this population is still relatively limited. Therefore, this study investigated children aged 6–12 years with flat feet, comparing them with children with normal arches regarding body image anxiety, social avoidance behaviour, depression symptoms, and self-worth. The study further aimed to analyse the association between flatfoot severity and these psychological variables, providing empirical evidence for the clinical psychological assessment and comprehensive intervention of children with flat feet.

## Materials and Methods

### General Information

This study conducted a retrospective analysis of the clinical data of 202 paediatric outpatients and inpatients (aged between 6 and 12 years) who visited Haiyan People’s Hospital Affiliated to Jiaxing University from January 2022 to June 2025. All data, including demographic characteristics, clinical assessments, and scale results, were extracted from the electronic medical record system.

Inclusion criteria were as follows: (1) aged 6–12 years; (2) diagnosed as flat feet or normal arches through the optical foot assessment system, with diagnostic results documented in the medical records; and (3) availability of complete medical records, including the results of the Body Image Scale (BIS), Social Anxiety Scale for Children (SASC), Children’s Depression Inventory (CDI), and Self-Perception Profile for Children (SPPC). Exclusion criteria were as follows: (1) previously psychological treatment or use of psychotropic drugs; (2) intellectual disability or developmental disorders; (3) other obvious physical abnormalities that may substantially affect body image or social behaviour, such as severe obesity or large-area birthmarks; and (4) previous foot surgery.

Assessment sequence: In this study, all psychological scales (BIS, SASC, CDI, SPPC) were assessed under uniform conditions. That is, the children were first diagnosed with flat feet or normal arches using an optical foot assessment system, and then all scales were assessed during the same outpatient visit.

### Research Methods

#### General Data Extraction

A standardized data extraction form was used to extract demographic characteristics and clinical information of the children from the medical records, including age, sex, school grade, parental education level, monthly household income, body mass index (BMI), severity of flat feet, gait abnormality (yes/no), limited motor ability (yes/no), history of interventions (e.g., foot orthotics, gait training), frequency of foot pain/fatigue, and whether they had received negative comments due to foot appearance or gait abnormalities. Data extraction was completed by two researchers independently to ensure accuracy.

#### Determination and Classification of Flat Feet

The classification of flat feet was based on the reports from the optical foot assessment system stored in the medical records. All classifications had been originally performed at the time of the patient’s visit by two trained researchers, with discrepancies reviewed by a senior physician. The arch was classified relative to a standard reference line. An arch that did not cross the line connecting the midpoint of the third toe to the centre of the heel was considered a normal arch; an arch located between the bisector of the angle between this line and the medial tangent was classified as mild flat feet; an arch located between the medial tangent and the bisector was considered moderate flat feet; and an arch crossing the medial tangent was considered severe flat feet. This grading standard was consistent with the study by Bahir *et al*. [12] and is illustrated in Fig. 1 (adapted from Figure 2 of Bahir *et al*. [12]).

**Fig. 1.**
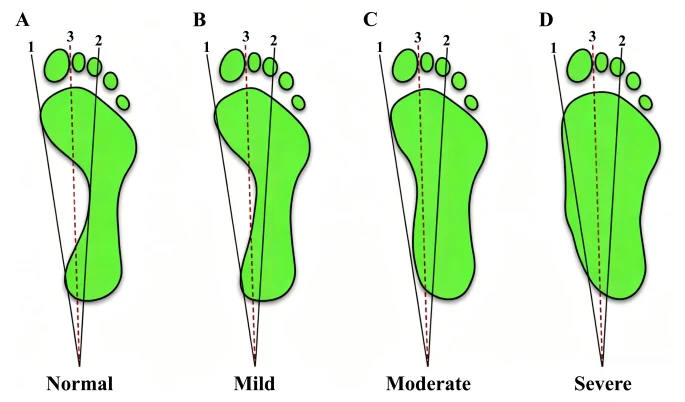
**Schematic diagram of the severity grading of flat feet based on footprint analysis. Adapted from Figure 2 of Bahir *et al*. (2026) [12], prevalence and modifiable risk factors for pediatric flatfoot among schoolchildren in Kunming and Kandahar: a cross-sectional study, published in Frontiers in Pediatrics (https://doi.org/10.3389/fped.2025.1739543). © 2026 Bahir *et al*. Licensed under the Creative Commons Attribution 4.0 International License (CC BY 4.0). The original footprint images were retained, and the annotation text was reformatted**. Note: The inner line from the big toe to the heel (L1) The straight line from the third toe to the center of the heel (L2); The angle bisector of the angle formed by L1 and L2 (L3). A flat foot arch not exceeding L2 is normal; between L2 and L3 is mild; between L3 and L1 is moderate; exceeding L1 is severe.

#### Assessment of Children’s Body Image Anxiety

Data on body image anxiety were obtained from archived psychological assessment records. The BIS was originally developed by Hopwood *et al*. [13] in 2001 to assess body image distress in adult cancer patients. However, the items on the scale, such as “I feel attractive” and “I am embarrassed about my body”, do not involve cancer-specific symptoms (e.g., hair loss, surgical scars) and have broad applicability. In recent years, the BIS has been explored for body image assessment in adolescents and people with physical deformities [14]. The scale covers three domains: emotional response, cognitive patterns and behaviour. It uses a 0–3 point four-point Likert scale with a total score of 0–30. Higher scores indicate greater psychological burdens.

This study included children aged 6–12 years with flat feet, and the assessment was administered as part of routine clinical assessment at the first visit. We made exploratory adaptations to the original scale: (1) replacing items such as “cancer treatment” and “surgical scars” with flatfoot-related items such as “foot appearance,” “walking posture,” and “shoe-wearing difficulties”; (2) simplifying the language, such as changing “I feel uncomfortable because of my body appearance” to “I feel uncomfortable because of the shape of my feet or my walking posture”; and (3) confirming the item comprehensibility through a pilot test with five target children. During the formal assessment, parents were permitted to assist younger children or children with reading difficulties by reading the questions and explaining the meaning of the items, but they were instructed not to interfere with the children’s choices.

Structural validity test: An exploratory factor analysis was conducted on this scale. The results showed that the Kaiser-Meyer-Olkin (KMO) measure of sampling adequacy was 0.86, and the cumulative variance explanation rate was 68.3%, indicating a good structural validity. Reliability test: The Cronbach’s α coefficient of the modified scale was 0.87, indicating good internal consistency.

#### Assessment of Children’s Social Avoidance Behaviour

Data on social avoidance behaviour were obtained from the SASC documented in the medical records. The scale, developed by La Greca *et al*. [15] in 1988, contains 10 items across two core dimensions—“fear of negative evaluation” and “social avoidance and distress”. The scale uses a 3-point rating method (0 = “never”, 1 = “sometimes”, 2 = “always”), yielding a total score range of 0–20 points. The score range for the fear of negative evaluation dimension is 0–12 points, and the score range for the social avoidance and distress dimension is 0–8 points. Higher total scores indicate more severe social anxiety and avoidance behaviours. For the purpose of this analysis, a total SASC score of >9 was defined as significant social anxiety and avoidance disorder, consistent with the threshold used in previous studies [16, 17].

#### Assessment of Childhood Depression Levels

Data on depressive symptoms were obtained from the CDI documented in the medical records. The scale was developed by Kovacs [18] in 1981 and is a widely used self-report depression screening tool for children worldwide. Based on the records, a shortened version with 12 items was administered [19]. Each item provides three descriptive options scored 0, 1, and 2 points, respectively. The total score ranges from 0 to 24 points, with higher scores indicating more severe depressive symptoms. In the extracted data, a total score of ≥10 points was considered indicative of clinically significant depressive symptoms [20], consistent with the original clinical assessment.

#### Assessment of Children’s Self-Worth

Data on self-worth were obtained from the SPPC documented in the medical records. The scale was developed by Harter [21] in 1985 and is applicable to children aged 8–14. It includes 36 items in six dimensions: academic ability, social acceptance, motor ability, appearance, behaviour and global self-worth. This study used six items from the global self-worth dimension. Each item consists of two contrasting statements. Children first chose the statement that best described them from two contrasting descriptions (e.g., “Some children are very satisfied with themselves,” but “Some children are not very satisfied with themselves”). They then judged whether the selected statement was “really true for me” or “sort of true for me.” Each item was scored on a four-point scale: 4 points for choosing a positive description and judging it as “really true for me,” and 3 points for “sort of true for me”; 2 points for choosing a negative description and judging it as “sort of true for me,” and 1 point for “really true for me.” The total score for the overall self-worth dimension ranged from 6 to 24 points, with higher scores indicating higher self-worth.

#### Quality Control

To ensure the accuracy of data extraction, the following quality control measures were implemented: (1) A standardized data extraction form was developed prior to data collection; (2) Two researchers independently extracted data from the medical records; (3) Discrepancies in data extraction were resolved through discussion with a third researcher; and (4) Records with more than 20% missing data on key variables were excluded from the analysis.

### Statistical Analysis

Statistical analyses were conducted using SPSS 26.0 (IBM Corp., Armonk, NY, USA). Continuous variables were assessed for normality using the Shapiro–Wilk test. Normally distributed variables were expressed as mean ± standard deviation, and comparisons between two groups were performed using an independent samples *t*-test. Variables not normally distributed were expressed as median (interquartiles), and comparisons were performed using the Mann–Whitney U test. Categorical variables were expressed as percentages (%) and analysed using the χ^2^ test. Spearman’s rank correlation analysis was used to analyse the correlation between the severity of flat feet, body image anxiety, social avoidance behaviour, depression level, and self-worth in children with flat feet. With the occurrence of significant social avoidance behaviour as the dependent variable and related factors as independent variables, a binary logistic regression model (stepwise forward method) was used to analyse influencing factors. In the single-factor analysis, to avoid missing potential important influencing factors due to the limited sample size, a more lenient significance level (α = 0.10) was adopted as the screening criterion for variables to enter the multivariable regression model. In the multivariable analysis, the significance level α = 0.05 (two-tailed), and *p* < 0.05 was considered statistically significant.

## Results

### General Information of Participants

This study included 202 participants, of whom 98 were in the flat-foot group and 104 were in the normal arch control group. There were no significant differences between the two groups in terms of age, sex, grade, parental education level, average monthly household income, BMI, or depression scores (*p* > 0.05), indicating comparable baseline characteristics. Compared with the control group, children with flat feet exhibited higher levels of body image anxiety and social avoidance behaviour, as well as lower self-worth (Table 1, Ref. [22, 23]).

**Table 1.** **General information of the study subjects [ n (%), $\bar{x}$ ± s ]**.

Project		Flatfoot group ( n = 98)	Normal arch group ( n = 104)	χ^2^ / *t*	*p*
Age (years)		9.17 ± 1.62	9.10 ± 1.57	0.344	0.731
Gender				0.001	0.980
	Male	52 (53.06)	55 (52.88)		
	Female	46 (46.94)	49 (47.12)		
Grade				0.472	0.790
	Lower grades	31 (31.63)	37 (35.58)		
	Middle grades	38 (38.78)	36 (34.61)		
	Upper grades	29 (29.59)	31 (29.81)		
Parents’ highest level of education				0.876	0.645
	High school and below	19 (19.39)	18 (17.31)		
	Associate’s/Bachelor’s degree	60 (61.22)	70 (67.31)		
	Master’s degree or above	19 (19.39)	16 (15.38)		
Average monthly household income per person (CNY)				1.432	0.231
	<3000	34 (34.69)	28 (26.92)		
	≥3000	64 (65.31)	76 (73.08)		
BMI (kg/m^2^)		17.53 ± 2.84	17.31 ± 2.49	0.594	0.553
Body image anxiety		16 .36 ± 5.21	11.75 ± 4.38	6.816	<0.001
Social avoidance behaviour		10.86 ± 3.37	7.23 ± 2.96	8.133	<0.001
Depression level		7.09 ± 3.64	6.79 ± 3.57	0.597	0.551
Self-worth level		14.69 ± 3.33	19.21 ± 2.69	10.570	<0.001

Note: Grade divisions [22] are based on the standard Chinese primary school education system: lower grades are grades 1–2, middle grades are grades 3–4, and upper grades are grades 5–6. The children included in this study were aged 6–12 years, all in primary school; no junior high school students were included. The household per capita monthly income is grouped based on the threshold of 3000 yuan. This threshold is determined by rounding down the average monthly disposable income per capita of residents nationwide in 2022, as published by the National Bureau of Statistics (approximately 3070 yuan, equivalent to 456 USD at the 2022 average exchange rate of 1 USD = 6.73 CNY) [23]. BMI, body mass index.

### Clinical Characteristics of the Flat Feet Group

Among the children in the flat-foot group, 42 cases (42.86%) had mild flat feet, 39 cases (39.79%) had moderate flat feet, and 17 cases (17.35%) had severe flat feet. The prevalence of gait abnormalities was 34.69%, and that of limited motor ability was 41.84%. Overall, 68.37% of the children reported never or rarely experiencing foot pain or fatigue, whereas 31.63% experienced related symptoms sometimes or frequently. 57.14% of the children had received interventions, such as orthotic insoles, and 42.86% had no history of intervention. Furthermore, 27.55% of the children had received negative feedback due to abnormal foot appearance or gait (Table 2).

**Table 2.** **Description of clinical characteristics of children with flat feet**.

Project		Frequency ( n )	Percentage (%)
Severity			
	Mild	42	42.86
	Moderate	39	39.79
	Severe	17	17.35
Gait abnormality			
	Yes	34	34.69
	No	64	65.31
Limited motor ability			
	Yes	41	41.84
	No	57	58.16
Pain/Fatigue frequency			
	Never/Rarely	67	68.37
	Sometimes/Often/Always	31	31.63
Previous intervention history			
	Yes	56	57.14
	No	42	42.86
Have you received negative feedback due to foot appearance or gait abnormalities?			
	Yes	27	27.55
	No	71	72.45

Note: Limited motor ability is defined as parents reporting that their child has significantly more difficulty than their peers when performing actions such as running, jumping, or standing on one leg, or as confirmed by a rehabilitation physician; The frequency of pain/fatigue “never/rarely” refers to no more than 1 time per month, while “sometimes/often/always” indicates 2 or more times per month or occurring every week; Previous intervention history includes the use of orthotic insoles, physical therapy (gait training, plantar muscle strengthening training), for at least 3 months.

### Harman’s Single-Factor Test

To eliminate the influence of common method bias on the results, we conducted a Harman single-factor test. We performed an unrotated principal component factor analysis on the item scores of all core variables in this study (body image anxiety, social avoidance behaviour, self-worth, depression). The results showed that the first unrotated factor explained 38.7% of the total variance, which was below the commonly used critical value of 40%, suggesting that the risk of common method bias was within an acceptable range.

### Correlation Analysis of Key Variables in the Flat Feet Group

Among children with flatfoot, disease severity exhibited strong positive correlations with body image anxiety (r = 0.886, *p* < 0.001) and social avoidance behaviour (r = 0.882, *p* < 0.001), alongside inverse associations with self-worth (r = –0.893, *p* < 0.001). Body image anxiety was positively correlated with social avoidance behaviour (r = 0.851, *p* < 0.001) and negatively with self-worth (r = –0.920, *p* < 0.001); social avoidance behaviour was also negatively correlated with self-worth (r = –0.822, *p* < 0.001) (Table 3).

**Table 3.** **Correlation analysis of key variables in children with flat feet**.

Variable	Severity		Body image anxiety		Social avoidance behaviour		Self-worth level	
	r	*p*	r	*p*	r	*p*	r	*p*
Severity	1	-						
Body image anxiety	0.886	<0.001	1	-				
Social avoidance behaviour	0.882	<0.001	0.851	<0.001	1	-		
Self-worth level	–0.893	<0.001	–0.920	<0.001	–0.822	<0.001	1	-

### Univariate Analysis of Factors Related to Social Avoidance Behaviours in Patients With Flat Feet

To further explore the correlation between body image anxiety and social avoidance behaviour in children with flat feet, significant social avoidance behaviour (defined as a total score of >9) among children with flat feet was set as the dependent variable. Demographic characteristics, clinical indicators related to flat feet, body image anxiety, depression level, and self-worth level were included as independent variables. Univariate logistic regression analyses were conducted using the stepwise forward method, with the inclusion criteria *p* < 0.10.

The assignment of independent variables is as follows: sex (male = 0, female = 1), grade (lower grades as the reference), parental education level (high school and below as the reference), monthly household income (<3000 yuan = 1, ≥3000 yuan = 0), abnormal gait (no = 0, yes = 1), limited motor ability (no = 0, yes = 1), frequency of pain/fatigue (never/rarely = 0, sometimes/often/always = 1), intervention history (no = 0, yes = 1), and negative comments related to foot appearance or abnormal gait (no = 0, yes = 1). Continuous variables (age, BMI, severity, body image anxiety, depression level, self-worth level) were all included in the analysis using their original values.

As shown in Table 4, univariate analysis revealed that the following variables were associated with significant social avoidance behaviour at *p* < 0.10: severity of flat feet (moderate: odds ratio (OR) = 2.892, 95% confidence interval (CI): 1.524–5.489, *p* = 0.001; severe: OR = 4.564, 95% CI: 2.109–9.877, *p* < 0.001), gait abnormality (OR = 2.342, 95% CI: 1.288–4.259, *p* = 0.005), limited motor ability (OR = 1.988, 95% CI: 1.098–3.601, *p* = 0.023), negative comments related to foot appearance or gait abnormalities (OR = 3.124, 95% CI: 1.614–6.048, *p* = 0.001), body image anxiety (OR = 1.231, 95% CI: 1.123–1.349, *p* < 0.001), and self-worth level (OR = 0.812, 95% CI: 0.742–0.889, *p* < 0.001). Demographic characteristics (age, sex, grade, parental education level, household income, BMI), pain/fatigue frequency, intervention history, and depression level were not significantly associated with social avoidance behaviour (*p* > 0.10). Variables with *p* < 0.10 were entered into the subsequent multivariable logistic regression model.

**Table 4.** **Univariate analysis of factors related to social avoidance behaviours in patients with flat feet**.

Variable		β	SE	Wald χ^2^	OR (95% CI)	*p*
Demographic characteristics						
Age		0.089	0.112	0.631	1.093 (0.877–1.362)	0.427
Gender		0.214	0.298	0.516	1.239 (0.691–2.221)	0.473
Grade						
	Lower grades	-	-	-	1	-
	Middle grades	0.312	0.341	0.837	1.366 (0.700–2.667)	0.360
	Upper grades	0.453	0.372	1.482	1.573 (0.759–3.259)	0.224
Parents’ highest education level						
	High school and below (reference)	-	-	-	1	-
	Associate’s/Bachelor’s degree	–0.187	0.389	0.231	0.829 (0.387–1.778)	0.631
	Master’s degree or above	–0.423	0.456	0.860	0.655 (0.268–1.601)	0.354
Monthly household income		–0.298	0.312	0.912	0.742 (0.402–1.369)	0.340
BMI (kg/m^2^)		0.067	0.058	1.334	1.069 (0.954–1.198)	0.248
Clinical characteristics						
Severity of flat feet						
	Mild (reference)	-	-	-	1	-
	Moderate	1.062	0.327	10.546	2.892 (1.524–5.489)	0.001
	Severe	1.518	0.394	14.842	4.564 (2.109–9.877)	<0.001
Gait abnormality		0.851	0.305	7.784	2.342 (1.288–4.259)	0.005
Limited motor ability		0.687	0.303	5.141	1.988 (1.098–3.601)	0.023
Pain/fatigue frequency		0.423	0.328	1.663	1.527 (0.803–2.904)	0.197
Previous intervention history		–0.312	0.301	1.075	0.732 (0.406–1.321)	0.300
Negative evaluations due to foot appearance or gait abnormalities		1.139	0.337	11.424	3.124 (1.614–6.048)	0.001
Psychological variables						
	Body image anxiety	0.208	0.047	19.584	1.231 (1.123–1.349)	<0.001
	Depression level	0.076	0.049	2.405	1.079 (0.980–1.188)	0.121
	Self-worth level	–0.208	0.046	20.444	0.812 (0.742–0.889)	<0.001

SE, standard error; OR, odds ratio; CI, confidence interval; BMI, body mass index.

### Multivariable Logistic Regression Analysis of Social Avoidance Behaviours in Children With Flat Feet

As shown in Table 5, multivariable regression analysis indicated that the severity of flat feet (with mild as the reference, moderate: OR = 2.399, 95% CI: 1.254–4.590, *p* = 0.008; severe: OR = 3.928, 95% CI: 1.766–8.738, *p* < 0.001), negative comments related to abnormal foot appearance or gait (OR = 2.683, 95% CI: 1.320–5.454, *p* = 0.006), and body image anxiety (OR = 1.172, 95% CI: 1.052–1.305, *p* = 0.004) were risk factors for significant social avoidance behaviour in children with flat feet; whereas self-worth level (OR = 0.845, 95% CI: 0.762–0.938, *p* = 0.002) was a protective factor. Notably, in the univariate analysis, abnormal gait and limited motor ability were significantly associated with social avoidance behaviour, but in the multivariable model, after adjusting for psychological variables, these factors no longer had significant associations. This result suggests that the association of gait abnormalities and limited motor skills and social avoidance behaviour may be mediated or attenuated by body image anxiety and self-worth levels.

**Table 5.** **Multivariable logistic regression analysis of social avoidance behaviours in children with flat feet**.

Variable		β	SE	Wald χ^2^	OR (95% CI)	*p*
Severity						
	Mild (reference)	-	-	-	1	-
	Moderate	0.875	0.331	6.987	2.399 (1.254–4.590)	0.008
	Severe	1.368	0.408	11.246	3.928 (1.766–8.738)	<0.001
Gait abnormality		0.412	0.342	1.451	1.510 (0.772–2.953)	0.228
Limited motor ability		0.298	0.335	0.791	1.347 (0.699–2.597)	0.374
Negative evaluations due to foot appearance or gait abnormalities		0.987	0.362	7.434	2.683 (1.320–5.454)	0.006
Body image anxiety		0.159	0.055	8.357	1.172 (1.052–1.305)	0.004
Self-worth level		–0.168	0.053	10.049	0.845 (0.762–0.938)	0.002
Constant		–2.016	1.019	3.914	0.133	0.048

SE, standard error; OR, odds ratio; CI, confidence interval.

## Discussion

This study focuses on the psychosocial adaptation problems of children with flat feet, systematically exploring the association and underlying mechanisms between body image anxiety and social avoidance behaviour. The results showed that children with flat feet exhibited higher levels of body image anxiety and social avoidance behaviour compared with children with normal arches, while also showing significantly lower self-worth level. Depressive symptoms did not differ significantly between the two groups. Correlation analysis further indicated a strong positive correlation between the severity of flat feet and body image anxiety and social avoidance behaviour, and a strong negative correlation with self-worth level. Logistic regression analysis showed that, after controlling for demographic characteristics and clinical variables, the severity of flat feet, negative comments related to foot appearance or gait abnormalities, and body image anxiety were risk factors for significant social avoidance behaviour in children with flat feet, whereas self-worth was a protective factor.

This study indicates that children with flat feet exhibit significant body image anxiety, and the severity of flat feet is highly positively correlated with body image anxiety. The reasons for this are twofold. Firstly, childhood is a critical period for the formation of body image, and awareness of appearance differences increases with age. For children with flat feet, changes in foot shape, gait differences, and functional limitations in some children may contribute to negative self-evaluations during peer comparisons [24, 25]. Furthermore, as a visible physical difference, the more severe the flat feet, the more obvious the change in appearance. According to the existing literature, any noticeable physical difference in childhood can attract peer attention or discussion, thereby exacerbating negative body perceptions among affected children [26, 27].

It is noteworthy that 27.55% of the children with flat feet in this study had experienced negative comments related to abnormal foot appearance or gait. This relatively high percentage suggests that external evaluations may play an important role in the formation of body image anxiety. Furthermore, there was no significant difference in depression levels between the flat feet group and the control group in this study. This negative result may be related to the following reasons: first, the psychological distress caused by flat feet may be mainly concentrated in specific areas (body image and social function) rather than diffuse mood disorders; second, the average depression scores of children in this study were all below the clinical threshold, suggesting that the overall mental health of the sample was acceptable and may not have reached the pathological level of depression.

This study found a significant increase in social avoidance behaviour among children with flat feet, as well as a strong correlation between body image anxiety and social avoidance behaviour. Further multivariate regression analysis showed that body image anxiety was a risk factor for social avoidance behaviour (OR = 1.172), indicating that higher levels of body image anxiety are associated with a higher risk of significant social avoidance behaviour. This finding may be interpreted within the framework of fear of negative evaluation: when individuals feel dissatisfied or ashamed of their physical characteristics, they may anticipate negative evaluations from others, leading to anxiety and social avoidance behaviours [28]. For children with flat feet, foot appearance or gait abnormalities can become a focal point of attention in social situations. To avoid potential ridicule, rejection, or excessive attention, children tend to withdraw, remain silent, or refuse to participate in group activities. Notably, this study found that negative comments related to foot appearance or gait abnormalities were a risk factor for social avoidance behaviour (OR = 2.683), with a stronger association than body image anxiety. This result suggests that negative social feedback experiences may directly reinforce children’s avoidance tendencies in social situations, with an impact that may even exceed the individual’s intrinsic anxiety experience.

This research has significant practical implications. For children with flat feet, clinical intervention should not be limited to orthotics and functional rehabilitation [29], but should also focus on their psychosocial adaptation. In particular, when carrying out visible interventions such as orthotic fitting, psychological support should be provided simultaneously to help children better understand their own condition, reduce stigma, and enhance self-worth [30]. Schools and families should also provide an inclusive environment to avoid rejection or bullying caused by physical differences.

This study has several limitations. First, the sample was drawn from a single hospital outpatient clinic, which may have introduced selection bias; future multi-centre studies are needed to confirm these findings. Second, causal relationships cannot be inferred from the cross-sectional design; longitudinal tracking studies are needed to clarify the temporal relationships between variables. Third, the study primarily relied on self-reporting scales; future studies could incorporate multi-dimensional indicators such as behavioural observation and peer evaluation. Fourth, the body image anxiety scale used in the study adjusted the original BIS scale in its expression. Although exploratory factor analysis initially supported its structural validity (KMO = 0.86, cumulative variance explained 68.3%), with good internal consistency (α = 0.87), this version has not yet been validated in independent samples. Therefore, the conclusions drawn based on this tool should be regarded as exploratory findings and should be interpreted with caution. Future research should adopt the standardized children’s BIS that has been cross-sample validated or combine it with semi-structured interviews for further validation. Fifth, the moderating role of environmental factors such as family support and peer relationships was not fully examined; these may play an important role in the psychological adaptation of children with flat feet.

## Conclusions

This study systematically describes the current status and association between body image anxiety and social avoidance behaviour in children with flat feet. It found that children with flat feet exhibited more pronounced body image anxiety and social avoidance behaviour than children with normal arches, and also had lower self-worth level. The severity of flat feet, experiences of negative evaluation, and body image anxiety were risk factors for social avoidance behaviour, whereas self-worth was a protective factor. In clinical practice, in addition to focusing on foot function rehabilitation, psychological assessment and intervention should also be incorporated. For example, psychological education can be introduced during orthopaedic treatment to help children correctly understand the prevalence and manageability of flat feet; anti-bullying education can be strengthened in the school to reduce peer rejection due to physical differences; and at the family level, parents should avoid overemphasizing abnormalities and instead reinforce their children’s overall strengths and sense of competence. Only by addressing both physical and mental well-being can the comprehensive healthy development of children with flat feet be truly promoted.

## Availability of Data and Materials

The datasets used and/or analysed during the current study were available from the corresponding author on reasonable request.
